# Delayed Port-Site Hematoma Following Robot-Assisted Hysterectomy Using the Hinotori Surgical Robot System in an Anticoagulated Patient With Cardiovascular Comorbidities: A Case Report

**DOI:** 10.7759/cureus.104354

**Published:** 2026-02-27

**Authors:** Yoshiko Kawata, Kenbun Sone, Yuichiro Miyamoto, Miyuki Harada, Yasushi Hirota

**Affiliations:** 1 Department of Obstetrics and Gynecology, The University of Tokyo Hospital, Tokyo, JPN

**Keywords:** endometrial cancer (ec), heparin bridging, port-site hematoma, robot-assisted surgery, superficial inferior epigastric artery flap

## Abstract

Perioperative management of patients on anticoagulation therapy requires careful balancing of the thromboembolic and bleeding risks. Heparin bridging is considered for high-risk patients; however, it may increase the incidence of postoperative bleeding complications. We report the case of a 74-year-old woman with stage IA endometrial cancer and cardiovascular comorbidities, including atrial fibrillation and mitral stenosis, who was receiving warfarin therapy. She underwent robot-assisted laparoscopic hysterectomy using the Hinotori surgical robot system with heparin bridging. On postoperative day 5, after restarting both heparin and warfarin, the patient developed a 9-cm subcutaneous hematoma at the right lower abdominal port site. Contrast-enhanced computed tomography suggested bleeding from a perforating branch of the inferior epigastric artery. Anticoagulation was interrupted, and local compression achieved hemostasis. After cautious reinitiation of anticoagulation therapy, the patient was discharged on postoperative day 14 without recurrent bleeding. This case highlights the importance of careful interpretation of activated partial thromboplastin time during heparin bridging in patients receiving warfarin, as additive anticoagulant effects may lead to excessive anticoagulation. In addition, preoperative imaging assessment of abdominal wall vasculature and careful port placement are essential to prevent delayed port-site hemorrhage during robot-assisted surgery.

## Introduction

With the aging population, the number of patients with cardiovascular diseases such as atrial fibrillation, ischemic heart disease, and heart failure who require antithrombotic therapy has increased [1]. As a result, surgeons increasingly encounter patients receiving anticoagulation therapy, making appropriate perioperative balancing of thromboembolic and bleeding risks a critical clinical challenge.

Endometrial cancer predominantly affects elderly women, and surgical resection remains the cornerstone of treatment. For early-stage disease, laparoscopic surgery has been widely adopted because of its minimally invasive nature, faster postoperative recovery, and shorter hospital stay and has been covered by Japan’s national health insurance system since 2014. Robot-assisted surgery for endometrial cancer was approved in Japan in 2018, allowing more precise surgical manipulation through stable three-dimensional visualization and tremor filtration.

Despite these advantages, port placement and robotic-arm design in robot-assisted surgery require careful consideration of their relationship with the abdominal wall vasculature from a safety perspective. In robot-assisted procedures, multiport placement and the absence of tactile feedback may make minor vascular injuries less apparent intraoperatively, increasing the risk of delayed port-site bleeding. This is particularly important in patients receiving anticoagulation therapy, in whom attention must be paid not only to intraoperative complications but also to the risk of delayed postoperative bleeding.

For the perioperative management of patients receiving anticoagulation therapy, heparin bridging is considered according to thromboembolic risk; however, it is associated with an increased risk of bleeding complications. The BRIDGE trial, reported in 2015, demonstrated that in patients with non-valvular atrial fibrillation, heparin bridging did not reduce thromboembolic events but significantly increased bleeding rates [2]. Importantly, this trial primarily included patients with non-valvular atrial fibrillation, and extrapolation of its findings to valvular atrial fibrillation should therefore be made with caution.

According to the 2022 Japanese Circulation Society and the Japanese College of Cardiology Guidelines for the Evaluation and Management of Cardiovascular Diseases in Non-Cardiac Surgery, perioperative heparin bridging is not recommended for patients at low-to-moderate thromboembolic risk, defined as a CHADS₂ (congestive heart failure, hypertension, age ≥ 75, diabetes, stroke (doubled)) score of 4 or below [3]. The CHADS₂ score is a clinical tool used to assess the risk of stroke in patients with atrial fibrillation, based on factors such as congestive heart failure, hypertension, age, diabetes, and prior stroke. In contrast, patients with non-valvular atrial fibrillation and a CHADS₂ score of 5 or 6, atrial fibrillation with moderate or greater mitral stenosis, pulmonary thromboembolism, cerebral infarction, or prosthetic heart valves are classified as high risk, and heparin bridging is recommended. Consistent with this, the European Society of Cardiology and the European Association for Cardio-Thoracic Surgery (ESC/EACTS) guidelines for the management of valvular heart disease classify atrial fibrillation associated with significant mitral stenosis as a high thromboembolic risk condition in which bridging anticoagulation may be considered when interruption of oral anticoagulation is necessary [4]. Nevertheless, for minimally invasive surgery (MIS), particularly robot-assisted surgery for gynecological malignancies, evidence regarding procedure-specific bleeding risks and the optimal timing for anticoagulant interruption and resumption remains limited, leaving decisions to individual institutional judgment.

Here, we report the case of an elderly woman with endometrial cancer, atrial fibrillation, and mitral stenosis receiving warfarin therapy who underwent robot-assisted hysterectomy using the Japanese-made Hinotori surgical robot system under heparin bridging and subsequently developed a delayed port-site hematoma.

## Case presentation

A 74-year-old woman (gravida 2, para 2) presented with abnormal genital bleeding. She was 148 cm tall, weighed 57.3 kg, and had a body mass index (BMI) of 26.2 kg/m². The subcutaneous tissue thickness at the planned port insertion site, measured on preoperative CT imaging, was approximately 2.5 cm. Her medical history included atrial fibrillation, mitral stenosis, hypertension, diabetes mellitus, and dyslipidemia. At 46 years of age, she underwent percutaneous mitral commissurotomy for symptomatic severe mitral stenosis. She had been followed by the cardiology department of another institution for atrial fibrillation, mitral stenosis, and hypertension and had been receiving warfarin at a dose of 3 mg daily.

Contrast-enhanced MRI did not reveal an obvious endometrial lesion; however, endometrial biopsy demonstrated grade 1 endometrioid carcinoma. Contrast-enhanced CT showed no evidence of distant metastases, and the patient was diagnosed with stage IA endometrial cancer. Owing to the need for careful perioperative management of her cardiovascular comorbidities and her preference for MIS, she was referred to our institution. After evaluation, the diagnosis of stage IA endometrioid carcinoma, grade 1, was confirmed, and robot-assisted laparoscopic hysterectomy was planned. Given the patient’s significant cardiovascular comorbidities, including atrial fibrillation and mitral stenosis, lymphadenectomy was omitted as part of the surgical plan. In the event of upstaging, adjuvant radiotherapy was planned as the follow-up treatment.

Given her high thromboembolic risk, the patient was admitted one week before surgery to initiate heparin bridging with unfractionated heparin (UFH). On admission, the baseline activated partial thromboplastin time (APTT) was 40 s (institutional reference range: 24-35 s). Although this value was already prolonged, likely reflecting warfarin effect, the target APTT was set at 60-80 s (1.5-2 times the admission value) in consideration of her cardiovascular risk following consultation with the cardiology team. Heparin was discontinued 8 h before surgery.

**Figure 1:**
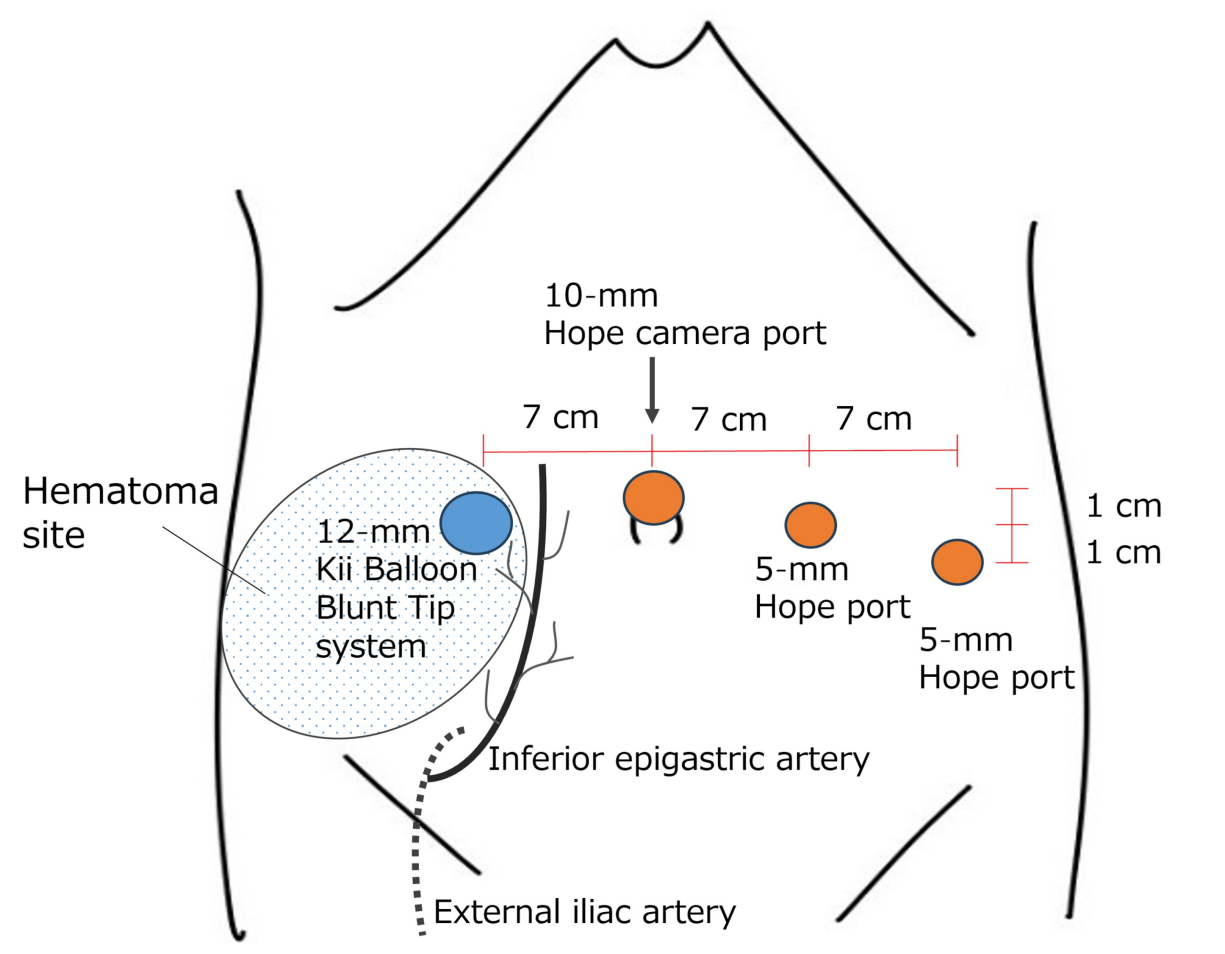
Schematic diagram of port placement, estimated course of the inferior epigastric artery, and its relationship to the abdominal wall hematoma The 10-mm camera port was placed above the umbilicus, with accessory ports arranged in a fan-shaped configuration at 7-cm intervals. The figure shows the estimated course of the inferior epigastric artery and the extent of the postoperative abdominal wall hematoma.

The procedure was performed using the Hinotori surgical robot system (Medicaroid Corporation, Kobe, Japan). A 10-mm camera port (Hope Corporation, Chiba, Japan) was placed above the umbilicus. Two 8-mm ports were inserted on the left side, 7 cm lateral to the umbilicus and 1 cm caudal to it, and an additional port was placed a further 7 cm lateral and 1 cm caudal. On the right side, a 12-mm Kii Balloon Blunt Tip system (Applied Medical Japan, Tokyo, Japan) was inserted 7 cm lateral to the umbilicus and 1 cm caudal, establishing a fan-shaped four-port configuration (Figure 1). No evidence of abdominal wall vascular injury or port-site bleeding was observed intraoperatively. The operative time was 3 h 32 min; the console time was 2 h 29 min; and the estimated blood loss was negligible and not quantitatively measured.

**Figure 2:**
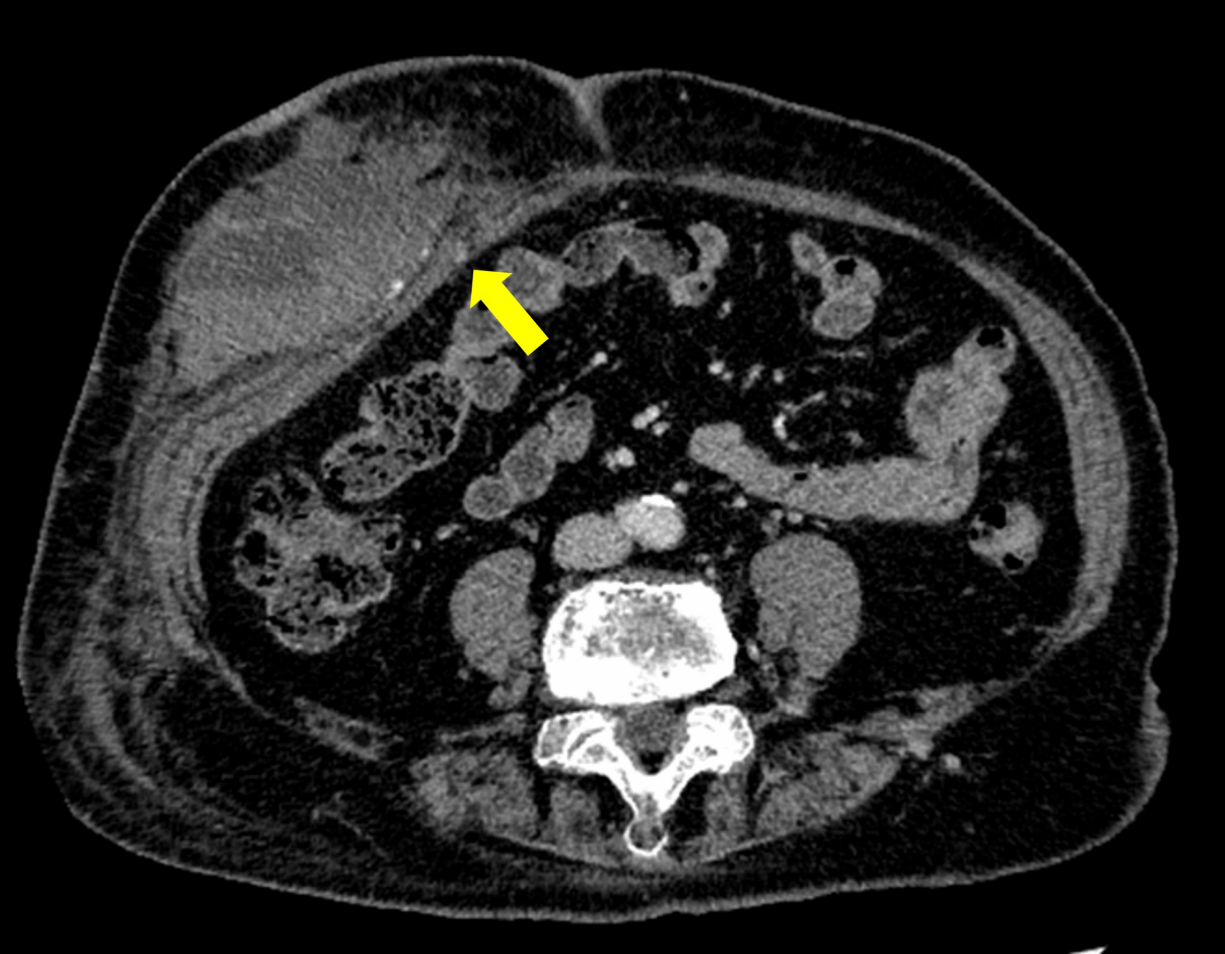
Contrast-enhanced CT showing bleeding from a perforating branch of the inferior epigastric artery

Heparin was resumed at 10,000 units/day 6 h postoperatively after confirming the absence of clinical signs of bleeding (prothrombin time-international normalized ratio (PT-INR), 1.02; APTT, 23.0 s). Heparin doses are expressed as 24-hour equivalent daily doses calculated from the infusion rate, even on days when the infusion was temporarily interrupted. Given her high thromboembolic risk and the minimally invasive nature of the procedure without lymphadenectomy, early resumption was considered appropriate once surgical hemostasis was secured. To mitigate postoperative bleeding risk, unfractionated heparin was restarted at a low intensity and subsequently titrated stepwise to 19,000 units/day under close monitoring. Warfarin 3 mg was restarted on postoperative day (POD) 4 (PT-INR, 1.09; APTT, 39.5 s), while therapeutic-dose unfractionated heparin (19,000 units/day) was continued as part of bridging anticoagulation therapy. On POD 5, laboratory testing revealed a PT-INR of 1.21 and an APTT of 57.3 s, and a subcutaneous hematoma measuring approximately 9 cm in maximum diameter was observed in the right lower abdomen. Hemoglobin levels dropped from 11.5 g/dL on POD 4 to 9.5 g/dL on POD 5. Anticoagulation was immediately discontinued, and compression of the abdominal wall was initiated. Contrast-enhanced CT suggested active bleeding from a perforating branch of the right inferior epigastric artery (Figure 2).

**Figure 3:**
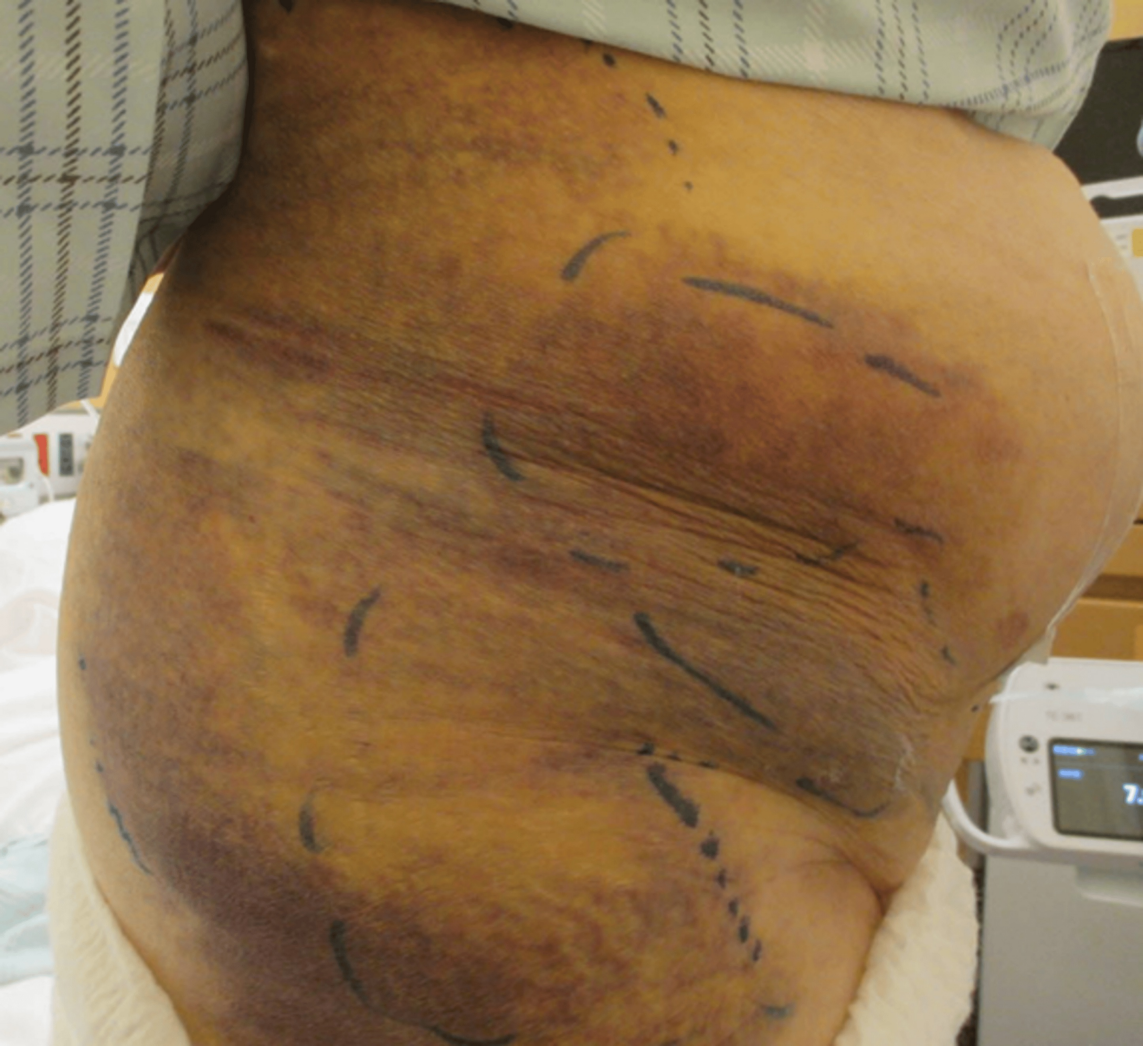
Subcutaneous hematoma three days after hemostasis

After confirming the absence of hematoma expansion by transabdominal ultrasonography (Figure 3), heparin was restarted at 5,000 units/day and gradually increased to 10,000 units/day.

**Figure 4:**
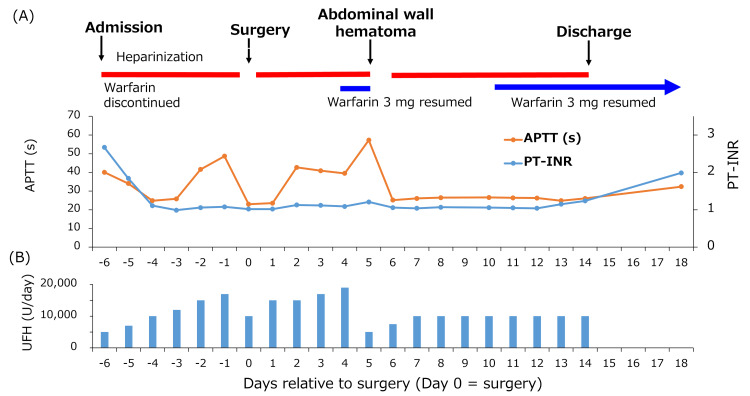
Timeline of anticoagulation management and changes in coagulation parameters during hospitalization (A) Clinical course with serial APTT and PT-INR values. (B) UFH dose (U/day; 24-hour equivalent based on infusion rate). Days are shown relative to surgery (Day 0). APTT: Activated partial thromboplastin time; PT-INR: Prothrombin time-international normalized ratio; UFH: Unfractionated heparin.

Warfarin (3 mg) was resumed on POD 10 (PT-INR, 1.06; APTT, 26.6 s), with no subsequent rebleeding. Heparin was discontinued on POD 14 (PT-INR, 1.24; APTT, 26.1 s), and the patient was discharged (Figure 4).

Follow-up contrast-enhanced CT at two and three months postoperatively confirmed resolution of the hematoma. The final pathological diagnosis was stage IB endometrial cancer, and as per the preoperative plan, adjuvant radiotherapy was administered based on the presence of deeper myometrial invasion. To date, there has been no recurrence.

Written informed consent was obtained from the patient for publication of this case report and any accompanying images.

## Discussion

This case illustrates a delayed port-site hematoma following robot-assisted surgery in an elderly patient with endometrial cancer receiving anticoagulation therapy. It provides relevant insights into perioperative anticoagulation management and highlights complications that may be specific to robot-assisted procedures. With population aging, the prevalence of cardiovascular comorbidities, such as atrial fibrillation, ischemic heart disease, and heart failure, has increased, leading to a growing number of patients receiving antithrombotic therapy [1]. At the same time, indications for MIS for gynecological malignancies have expanded, increasing the importance of perioperative anticoagulation management.

Although the use of warfarin has declined with the widespread adoption of direct oral anticoagulants (DOACs), it remains the first-line therapy for patients with valvular atrial fibrillation, mechanical heart valves, severe renal impairment, or antiphospholipid syndrome. Warfarin exerts its anticoagulant effect by inhibiting the gamma-carboxylation of vitamin K-dependent coagulation factors (II, VII, IX, and X). As factor VII has the shortest half-life, the PT-INR is primarily used to assess the anticoagulant effect; however, APTT may also be prolonged due to reductions in factors IX and X [5].

In the present case, APTT prolongation was already present during preoperative warfarin therapy. At admission, the baseline APTT was 40 s, exceeding our institutional reference range (24-35 s), suggesting residual warfarin-associated prolongation. Without sufficient consideration of this effect when determining the perioperative heparin dose, the additive anticoagulant effects of unfractionated heparin after warfarin resumption may have resulted in excessive anticoagulation. It is well established that during combined warfarin and unfractionated heparin therapy, the anticoagulant effects of both agents are additive, which can result in over-prolongation of APTT [6]. In addition, setting the heparin therapeutic target as 1.5-2 times an already prolonged baseline value may have predisposed the patient to supratherapeutic anticoagulation during the perioperative period. In this patient, the further prolongation of APTT following postoperative warfarin resumption suggests that this additive effect became clinically significant. However, because the PT-INR remained subtherapeutic (1.21) when APTT increased sharply from 39.5 s to 57.3 s within one day, this rapid elevation was likely driven predominantly by ongoing therapeutic-dose heparin (19,000 units/day) rather than by the full anticoagulant effect of warfarin.

In retrospect, closer reassessment and earlier down-titration of heparin during overlap therapy, before or at the time of warfarin resumption, might have reduced the risk of excessive anticoagulation and delayed bleeding. When APTT prolongation is observed, careful evaluation is required to distinguish the contribution of warfarin from that of heparin, and measurement of anti-Xa factor activity or thrombin time may be useful [7]. Such additional assays may be particularly helpful when baseline APTT is prolonged before heparin initiation, as APTT-based titration alone can overestimate heparin requirements and obscure the relative contributions of each anticoagulant. In elderly patients, altered drug metabolism due to hepatic and renal dysfunction or hypoalbuminemia may further enhance anticoagulant effects even at low doses, necessitating more stringent monitoring. We acknowledge that anti-Xa activity was not measured in this case. Therefore, the additive anticoagulant effect was inferred primarily from APTT trends in the context of subtherapeutic PT-INR levels, which represents a limitation of this report.

In minimally invasive laparoscopic surgery, multiport placement and manipulation under pneumoperitoneum can make minor intraoperative bleeding difficult to visualize, particularly at port sites, which can subsequently manifest as delayed postoperative hematoma or hemorrhage [8]. Major abdominal wall vessels include the superficial epigastric, superficial circumflex iliac, deep inferior epigastric, and deep circumflex iliac arteries running beneath the rectus abdominis muscle. Although superficial vessels can be visualized by transillumination, deeper vessels such as the inferior epigastric artery and deep circumflex iliac artery are often difficult to visualize intraoperatively. The incidence of abdominal wall vascular injury during laparoscopic surgery is reported to be less than 2%, with most injuries involving the inferior epigastric artery and its branches [9]. The main trunk of the inferior epigastric artery typically runs 4-8 cm lateral to the midline; however, in elderly or obese patients, its course may deviate further laterally. Therefore, when placing secondary and tertiary ports in cases where the abdominal wall vessels are difficult to visualize, awareness of the risk of injury to the main trunk or perforating branches of the inferior epigastric artery is essential [10]. In this case, imaging findings suggested injury to a perforating branch of the inferior epigastric artery, and conservative management with compression and anticoagulation adjustment achieved hemostasis. However, in cases of refractory bleeding, reoperation or transcatheter arterial embolization (TAE) may be required [11].

Our institution introduced the da Vinci Xi Surgical System (Intuitive Surgical, Inc., Sunnyvale, CA) in 2018 and added the Hinotori surgical robot system (Medicaroid Corporation, Kobe, Japan) in November 2023. Horizontal port placement above the umbilicus is commonly used with the da Vinci system, whereas the Hinotori system employs a fan-shaped port configuration centered on the camera port to minimize robotic-arm interference, which was adopted in this case. Previous studies have reported no significant differences in complication rates between robotic platforms used in robot-assisted gynecological surgery [12,13]. Nevertheless, because port placement concepts and configurations differ among robotic systems, their relationship to abdominal wall vasculature is not necessarily equivalent. As the inferior epigastric artery courses superomedially from the inguinal region, particular caution is required when placing ports in more caudal or lateral positions. Although the Hinotori system features a docking-free design that reduces mechanical stress on the abdominal wall [14], it remains speculative whether this leads to a clinically meaningful reduction in port-site vascular injury risk. Even minor vascular injuries can become clinically significant postoperatively in patients receiving anticoagulation therapy.

This case suggests that vascular injury near port insertion sites may result in postoperative complications, particularly in patients receiving anticoagulation therapy. The patient had undergone contrast-enhanced CT for staging purposes, yet abdominal wall vascular anatomy was not specifically assessed in this study.

In robot-assisted surgery, careful consideration of port placement is essential. Preoperative contrast-enhanced CT may provide useful anatomical information regarding the course of the inferior epigastric artery and its proximity to the midline. However, the routine use of preoperative vascular assessment and its clinical impact have not yet been established.

## Conclusions

This case highlights that APTT monitoring during heparin bridging after temporary interruption of warfarin can be confounded by residual warfarin effects, particularly during overlap therapy, potentially leading to excessive anticoagulation if interpreted in isolation. In such settings, anti-factor Xa activity assays may offer a more specific assessment of heparin effect when APTT interpretation is uncertain. Furthermore, even minor abdominal wall vascular injuries at port sites can manifest as delayed hematoma formation in anticoagulated patients undergoing robot-assisted surgery. Careful anticoagulant titration, meticulous port-site hemostasis, and preoperative assessment of abdominal wall vascular anatomy are essential to enhance procedural safety.
